# Supplementary material for: WordCommentsAnalyzer: A windows software tool for qualitative research
Source: F1000Res. 2018 Sep 4;7:536. Originally published 2018 May 3. [Version 2] doi: 10.12688/f1000research.14819.2 (PMC6137411; doi:10.12688/f1000research.14819.2)
Supplement: Supplementary file 3 [file f1000research-7-17614-s0002.tgz › b2a79496-4ada-47c4-9682-a99ce867836d.zip!/Eight Iranian Health Journals/Yafteh _17.docx]

© Yafteh  . This work is licensed under CC BY 4.0 (https://creativecommons.org/licenses/by/4.0/).

Volume & Issue: دوره 16 - شماره 1

Title: بررسی مقایسه ای روش های آزمون سریع و روتین در سنجش نشانگرهای قلبی در بیماران مشکوک به حمله قلبی حاد

http://yafte.lums.ac.ir/article-1-1582-fa.html

مقدمه: در بسیاری از بیماران با درد حاد قفسه سینه، تشخیص صحیح سکته حاد قلبی وابسته به نشانگرهای قلبی نظیر ایزوآنزیم کراتین کیناز قلبی(CK-MB) و تروپونین I قلبی (cTnI)است چرا که الکتروکاردیوگرام در اغلب موارد غیرتشخیصی است. اندازه گیری نشانگرهای قلبی برای تعیین صدمه کوچک عضله قلب مقدم هستند و صحت تشخیصی بیشتری دارند. هدف از این مطالعه ارزیابی روش سنجش روتین CK-MB و cTnI در قیاس با روش تست سریع در بیماران مشکوک به سکته حاد قلبی می باشد. بحث و نتیجه‌گیری: نتایج حاضر نشان داد که تست سریع cTnI جهت تشخیص سکته حاد قلبی بسیار اختصاصی بوده ولی حساسیت پایینی دارد در حالیکه تست سریع CK-MB جهت تشخیص سکته حاد قلبی در بیماران با درد حاد قفسه سینه هم حساسیت و هم اختصاصیت پایینی دارد. یافته‌ها: نتایج سنجش الایزا cTnI بیماران 52 مثبت و 90 منفی در قیاس با سنجش تست سریع cTnIکیتGolden 27 مثبت و 110 منفی، در حالیکه در قیاس با سنجش تست سریع cTnIکیت Acon بیماران 30 مثبت و 112 منفی می باشد)001/0(P≤ نتایج سنجش فتومتری CK-MB بیماران 39 مثبت و 103 منفی در قیاس با سنجش تست سریع CK-MB کیتGolden 60 مثبت و 77 منفی، در حالیکه در قیاس با سنجش تست سریع CK-MB کیت Acon بیماران 78 مثبت و 64 منفی می باشد) 035/0P≤) حساسیت نسبی و ویژگی نسبی در قیاس با روش های روتین برای تست سریع cTnIکیتGolden بترتیب 50% و 8/98%، برای تست سریع cTnI بترتیب کیت Acon9/51% و 6/96، برای تست سریع CK-MB کیتGolden بترتیب 2/69% و 1/63% و برای تست سریع CK-MB کیت Acon بترتیب 2/69% و 4/50% می باشد. مواد و روش‌ها: افراد شرکت کننده در این مطالعه 142 بیمار با درد حاد قفسه سینه با شروع درد کمتر از 12 ساعت بستری شده در بخش اورژانس بودند. نمونه خون وریدی جهت سنجش تست های سریع CK-MB و cTnI با کیت های Acon و Golden و اندازه گیری cTnI با الایزا و CK-MB با فتومتری گرفته شد.

Title: بررسی سطح سرمی IgG علیه هلیکوباکترپیلوری در کودکان 1 تا 14 ساله مراجعه کننده به بیمارستان کودکان شهید مدنی در سال 91-1390

http://yafte.lums.ac.ir/article-1-1583-fa.html

مقدمه: هلیکوباکتر پیلوری یکی از عوامل عفونت‌زای انسانی است که باعث ایجاد گاستریت و زخم پپتیک می‌شود و با بدخیمی معده در ارتباط می‌باشد. با توجه به نقش غیرقابل انکار این باکتری در ایجاد ناراحتی‌های سیستم گوارشی و فقدان اطلاعاتی در خصوص شیوع این عفونت درکودکان 1 تا 14 ساله استان لرستان، این مطالعه با هدف تعیین شیوع عفونت سرمی هلیکوباکتر پیلوری در مراجعین به بیمارستان کودکان شهید مدنی شهر خرم‌آباد و بررسی فاکتورهای خطر مرتبط با آن انجام شد. بحث و نتیجه‌گیری: بر اساس مقادیر سرمی آنتی بادی هلیکوباکتر پیلوری؛ شیوع این عفونت با سن، قد و وزن فعلی کودک ارتباط خطی مستقیم وجود دارد (05/0>P). به این معنا که با سن و قد و وزن کودک مقادیر آنتی بادی سرمی عفونت هلیکوباکتر پیلوری افزایش معناداری نشان داد. همچنین فراوانی عفونت هلیکوباکتر پیلوری در خانواده های با درآمد کمتر از 250 هزار تومان، به طور معنی دار، بالاتر از کودکان سایر خانواده ها بود. یافته‌ها: در این مطالعه تعداد 200 کودک زیر 14 سال از نظر فراوانی عفونت هلیکوباکتر پیلوری مورد بررسی قرار گرفتند. میانگین سنی کودکان 9/2±1/5 سال بود. 61% کودکان پسر و 39% دختر بودند. بر اساس سن، بیشترین فراوانی در گروه سنی 14-10 سال (9/57%) و کمترین فراوانی در گروه سنی زیر 1 سال (7/16%) بود و بر اساس آزمون دقیق فیشر؛ معنادار بود (032/0=P). فراوانی عفونت هلیکوباکتر پیلوری در خانواده های با درآمد کمتر از 250 هزار تومان بالاتر از کودکان سایر خانواده ها بود ( 2/54%) و بر اساس آزمون کای اسکوئر؛ این اختلاف به لحاظ آماری معنادار بود (006/0=P). بر اساس نتایج این مطالعه تفاوتی در فراوانی عفونت هلیکوباکتر پیلوری در کودکان ساکن شهر و روستا دیده نشد. مواد و روش‌ها: مطالعه حاضر از نوع اپیدمیولوژیک تحلیلی بوده که درسال 91-1390 انجام شد. بدین منظور با والدین کلیه بیماران مراجعه کننده به کلینیک ویژه بیمارستان کودکان مدنی خرم آباد مصاحبه شد و پس از تکمیل پرسشنامه و اخذ رضایتنامه کتبی، کودکان جهت اخذ نمونه به آزمایشگاه ارجاع شدند. سپس سطح سرمی IgG علیه هلیکوباکترپیلوری بیماران به روش الایزا تعیین و در پرسشنامه وارد گردید. اطلاعات دموگرافیک نظیر سن، جنس، سطح تحصیلات مادر، شغل پدر نیز وارد پرسشنامه شد سپس تجزیه و تحلیل آماری با نرم افزارSPSS انجام گرفت.

Title: بررسی کارآیی فرآیند اکسیداسیون پیشرفته UV/H2O2 در حذف آنتی‌بیوتیک ‌سفتریاکسون از محیط آبی

http://yafte.lums.ac.ir/article-1-1584-fa.html

مقدمه: حضور آنتی‌بیوتیک‌ها در منابع آبی، نشانگر ناکارآمدی فرآیندهای متداول تصفیه در حذف آ‌ن‌ها می‌باشد. هدف از این مطالعه بررسی اثربخشی روش اکسیداسیون پیشرفته UV/H2O2 در حذف سفتریاکسون از محیط آبی در مقیاس آزمایشگاهی است. مواد و روش‌ها: این تحقیق یک مطالعه تجربی است که در مقیاس آزمایشگاهی و در راکتوری با حجم 5800 میلی لیتر انجام شده است. محلول آبی حاوی آنتی‌بیوتیک سفتریاکسون با غلظت‌های 9، 18 و 27 میلی‌گرم در لیتر ساخته شد. اثر غلظت اولیه آنتی‌بیوتیک، pH، زمان ماند، ولتاژهای مختلف لامپUV و غلظت H2O2 بر راندمان حذف این آلاینده از محیط آبی مورد مطالعه قرار گرفت. برای اندازه گیری غلظت سفتریاکسون از HPLC استفاده شد. بحث و نتیجه‌گیری: روش اکسیداسیون پیشرفته با استفاده از UV/H2O2 در شرایط مناسب محیطی قادر به حذف مؤثر آنتی بیوتیک سفتریاکسون از محیط‌های آبی می‌باشد. یافته‌ها: نتایج حاصل از این مطالعه نشان داده است که روش UV/H2O2قادر به حذف 75% سفتریاکسون از محلول آبی حاوی 9 میلی‌گرم در لیتر، با زمان ماند 3 ساعت می‌باشد. میزان حذف سفتریاکسون متأثر از پارامترهای pH، غلظت اولیه سفتریاکسون، غلظت H2O2 و شدت تابش اشعه UV می‌باشد. به طوری‌که در pHهای اسیدی (pH برابر 3) و غلظت پایین سفتریاکسون (9 میلی گرم بر لیتر)، غلظت H2O2 برابر 15 میلی گرم در لیتر و شدت تابش بالا (36 وات) راندمان بالاتری در حذف آنتی بیوتیک حاصل شده است.

Title: اثربخشی روان درمانی گروهی امید محور بر بهبود ابعاد کیفیت زندگی مرتبط با سلامت در بیماران مبتلا به سرطان

http://yafte.lums.ac.ir/article-1-1585-fa.html

مقدمه: امید از عوامل بسیار مهم و از عناصر ضروری در بیماران سرطانی است که اثرات زیادی در سازگاری بیماران با شرایط زندگی، به خصوص در زمان درد و محرومیت دارد. امید در افزایش کیفیت زندگی بیمار سهم به سزایی داشته و در مراحل مختلف بیماری تاثیرگذار می باشد. هدف از این مطالعه بررسی اثربخشی مداخلات ارتقاء دهنده امید در بیماران مبتلا به سرطان بود. بحث و نتیجه‌گیری: مداخلات ارتقاء دهنده امید در افزایش امید بیماران می باشد. بنابراین بجاست که به منظور افزایش کیفیت زندگی بیماران مبتلا به سرطان، ارائه خدمات آموزشی و برنامه های ارتقاء دهنده امید با روشهای مناسب مانند یادگیری بر اساس حل مسئله یا مشارکت فعال بیمار در فرایند بحث با برنامه ریزی مدون انجام گیرد. یافته‌ها: تحلیل داده ها نشان داد که بین میانگین نمره های پس آزمون گروه آزمایش و کنترل تفاوت معنی داری وجود داشت(01/0< P)، به نحوی که آموزش روان درمانی گروهی امید محور موجب بهبود ابعاد کیفیت زندگی مرتبط با سلامت در گروه آزمایش شده است. مواد و روش‌ها: این مطالعه یک کارآزمایی بالینی بود که در سال 1390، در بیمارستان شفای اهواز بر روی 30 نفر از بیماران مبتلا به سرطان انجام شد. قبل و بعد از مداخله برای هر دو گروه آزمایش و کنترل پرسشنامه کیفیت زندگی مرتبط با سلامت تکمیل شد (هر گروه 15 بیمار). مداخلات ارتقاء دهنده امید برای بیماران گروه آزمایش به صورت مباحثه عمیق و چهره به چهره و با روش یادگیری بر اساس حل مسئله انجام شد. داده ها با استفاده از تحلیل کوواریانس چند متغیره مورد تجزیه و تحلیل قرار گرفتند.

Title: بررسی اثر کلونیدین خوراکی به صورت پیش دارو قبل از بیهوشی بر روی میزان خونریزی حین عمل تمپانوپلاستی

http://yafte.lums.ac.ir/article-1-1586-fa.html

مقدمه: خونریزی حین اعمال جراحی یکی از مهمترین عوارض در طی انواع جراحی هاست. جهت کاهش این مشکل تکنیک‌ها و روش‌های مختلفی استفاده شده است. در این مطالعه، اثر پیش داروی کلونیدین خوراکی در ایجاد محدوده عمل تا حد امکان عاری از خون در بیماران تحت عمل تمپانوپلاستی را مورد بررسی قرار دادیم . بحث و نتیجه‌گیری: پیش درمانی با کلونیدین میزان خونریزی حین عمل تمپانوپلاستی را کاهش و میزان رضایتمندی جراح را افزایش می دهد. یافته‌ها: هیچ اختلاف آماری معناداری بین جنس و سن در دو گروه وجود نداشت. تغییرات همودینامیک در گروهی که کلونیدین دریافت کرده بودند نسبت به گروه شاهد کمتر بود. میزان خونریزی در گروهی که داروی کلونیدین دریافت کرده بودند 6/54 برابر نسبت به گروه شاهد کمتر و رضایتمندی جراح نیز 6/54 برابر در گروه کلونیدین بیشتر از گروه شاهد بود. تجزیه و تحلیل آماری با استفاده از آزمون T مستقل، تفاوت آماری معنی داری بین دو گروه نشان داد. مواد و روش‌ها: این مطالعه به صورت کارآزمایی بالینی سه سوکور تصادفی روی 88 بیمار کاندید عمل جراحی تمپانوپلاستی انجام گردید. بیماران به صورت تصادفی به دو گروه تقسیم شدند. 44 بیمار 90 دقیقه قبل از رسیدن به اتاق عمل 300 میکروگرم کلونیدین و 44 بیمار دارونما دریافت کردند. روش بیهوشی در هر دو گروه یکسان بود. خونریزی حین عمل با استفاده از یک سیستم چهارتایی از 0 = بدون خونریزی تا III = خونریزی شدید ارزیابی شد. رضایتمندی جراح از محدوده عمل جراحی و تغییرات همودینامیک بررسی و در پرسشنامه ثبت شدند.

Title: بررسی اثر شیمی درمانی کمکی در مرحله IIکانسرهای کولورکتال

http://yafte.lums.ac.ir/article-1-1587-fa.html

مقدمه: سرطان‌های کولورکتال بدخیم ترین و شایعترین سرطان دستگاه گوارش است که چهارمین سرطان شایع در سراسر دنیا می باشد. انتخاب نوع روش درمانی براساس مرحله بندی بیماری انجام می شود. در این پژوهش برآنیم تا با انجام مطالعه مداخله‌ای اثر شیمی درمانی کمکی را در میزان عود موضعی، متاستاز دوردست و نیز طول عمر 3 ساله عاری از بیماری را در مبتلایان به کانسر مرحله II کولورکتال بررسی کنیم. مواد و روش‌ها: در این مطالعه 40 فرد مبتلا به کانسرهای کولورکتال مرحله II از سال 86-1380 وارد مطالعه شدند که پس از انجام جراحی ، شیمی درمانی ادجوانت طبق رژیم درمانی FOLFOX4 برای آنها انجام شد و هر 3 ماه با استفاده از تومور مارکر، سونوگرافی و سی تی اسکن و نیز کولونوسکوپی از نظر عود موضعی و متاستاز بررسی گردیدند. نهایتاً در پایان سال سوم پس از شیمی درمانی میزان عود موضعی، متاستاز و بقای بیماران بررسی شد. بحث و نتیجه‌گیری: در این مطالعه شیمی درمانی کمکی درمرحله II کانسرکولورکتال باعث بقا کلی و بقا عاری از بیماری 3 ساله قابل توجهی شده است. یافته‌ها: در این پژوهش 40 بیمار مورد مطالعه قرارگرفتند که 23 نفر مرد و مابقی زن بودند. پس از 3 سال پیگیری، 2 نفر عود موضعی ، 7 نفر متاستاز دوردست داشتند. 31 نفر از افراد مورد مطالعه پس از 3 سال پیگیری نه دچار عود موضعی شدند و نه متاستاز دوردست داشتند ، به عبارت دیگر طول عمر 3 ساله عاری از بیماری 5/77% بود.

Title: شناسایی رابطه بین هوش معنوی و سلامت روانی دانشجویان دانشگاه علوم پزشکی لرستان

http://yafte.lums.ac.ir/article-1-1588-fa.html

مقدمه: مسائل مربوط به سلامت روان دانشجویان در سال‌های اخیر توجه زیادی را به خود معطوف کرده است. عوامل مختلفی در سلامت ‌روان نقش دارند . امروزه معنویت به عنوان یک جنبه‌ مهم از کنش انسان­گرایانه، که رابطه پایایی با سلامت و بهبودی دارد مورد توجه قرار گرفته است. لذا هدف اصلی این پژوهش شناسایی رابطه بین هوش معنوی و سلامت روانی دانشجویان دانشگاه علوم پزشکی استان لرستان بوده است.   بحث و نتیجه­گیری : هوش معنوی تاثیر مثبتی در سلامت روانی افراد دارد. نتایج تحقیقات نشان می‌دهد افراد دارای زندگی معنوی، از نظر روان‌شناختی افراد سالم‌تری هستند.   یافته­ها: نتایج نشان داد که بین هوش معنوی و ابعاد سلامت روانی(«جامع‌نگری و بعد اخلاقی»، ‌«توانایی مقابله و تعامل با مشکلات»، «‌سجایای اخلاقی» و «خودآگاهی و عشق و علاقه») رابطه مثبت وجود دارد، همچنین ابعاد «خود‌آگاهی، عشق و علاقه» و «جامع‌نگری» سهم عمده‌ای در پیش‌بینی سلامت روانی دارند.   مواد و روش­ها: در این پژوهش از روش توصیفی از نوع همبستگی استفاده شد. جامعه آماری مورد مطالعه کلیه دانشجویان دانشگاه علوم پزشکی استان لرستان به تعداد 2238 نفر بودند. برای تعیین حجم نمونه از فرمول کوکران استفاده گردید و تعداد 330 دانشجو به عنوان نمونه‌ به روش نمونه‌گیری طبقه‌ای نسبی تصادفی انتخاب گردیدند. ابزار گردآوری داده‌ها دو پرسشنامه«سلامت روانی ( GHQ-28 ) گلدبرگ» و «هوش معنویِ بدیع و همکاران»، بود. اعتبار ابزار از طریق روایی محتوایی و پایایی پرسشنامه‌ها با استفاده از روش آلفای کرونباخ تعیین گردید. برای تجزیه و تحلیل داده‌ها و آزمون فرضیه‌های تحقیق علاوه بر شاخص‌های آمار توصیفی از جمله میانگین و انحراف معیار از آزمون‌های آمار استنباطی مانند ضریب همبستگی پیرسون و تحلیل رگرسیون چندگانه و آزمون t مستقل استفاده شده است.

Title: بررسی اثر تجویز بتائین خوراکی بر کیفیت اسپرم موش رت متعاقب مسمومیت با کادمیوم

http://yafte.lums.ac.ir/article-1-1589-fa.html

مقدمه: هدف از انجام این مطالعه، بررسی اثر حفاظتی بتائین خوراکی در برابر اثرات کادمیوم بر کیفیت اسپرم شامل حرکات پیشرونده اسپرم، سلامت غشاء اسپرم، غلظت و نیز وزن بیضه می باشد. مواد و روش ها: تعداد 30 عدد موش رت نر بالغ به 3 گروه 10 تایی شامل گروه کنترل-سالین، کادمیوم-سالین و کادمیوم-بتائین تقسیم شده و به دو گروه آخر یک دوز کادمیوم از طریق صفاقی تزریق شده و سپس به گروه سوم، از یک روز قبل از تزریق کادمیوم به مدت 10 روز پیاپی محلول بتائین خورانده شد. سپس 5 موش از هر 3 گروه در روزهای 5 و 10 پس از تزریق کادمیوم کشته شده و نمونه اسپرم از دم اپیدیدیم جهت ارزیابی آن اخذ شد. بحث و نتیجه گیری: بتائین احتمالاً به دلیل خاصیت آنتی اکسیدانی می تواند اثرات استرس اکسیداتیو ناشی از کادمیوم را در بافت بیضه کاهش دهد که این امر باعث افزایش سلامت غشاء اسپرم و متعاقباً افزایش نسبی حرکات پیشرونده اسپرم می گردد. یافته ها: کادمیوم به طور معنی داری باعث کاهش وزن بیضه ها شد. از طرف دیگر، درصد اسپرم های با حرکت پیشرونده و همچنین درصد اسپرم های با غشاء سالم و نیز غلظت اسپرماتوزوئیدها به طور معنی داری در گروه کادمیوم نسبت به گروه کنترل در هر دو روز کشتار کاهش یافت. درحالی که تجویز بتائین توانست درصد سلامت غشاء اسپرم را بطور معنی داری نسبت به گروه کادمیوم سالین در روز 10 افزایش دهد (8/28% در گروه بتائین در برابر 4/9% در گروه کادمیوم(044/0=P). اگرچه درصد حرکات پیشرونده اسپرم در روز 10 متعاقب تجویز بتائین نسبت به گروه کادمیوم-سالین افزایش داشت، اما این تفاوت از نظر آماری معنی دار نبود.

Title: بررسی تأثیر استویوساید و استویا ریبودیانا بر رشد استرپتوکوکوس موتانس

http://yafte.lums.ac.ir/article-1-1590-fa.html

مقدمه: استرپتوکوکوس موتانس عامل اصلی پوسیدگی دندان و تخریب آن است. هدف از این تحقیق بررسی آزمایشگاهی اثر استویوساید و عصاره های گیاه استویا ریبودیانا بر روی رشد سویه مورد نظر بود.   بحث و نتیجه­گیری : عصاره های الکلی و استونی گیاه استویا ریبودیانا می تواند به عنوان دارو برای پیشگیری و کنترل رشد استرپتوکوکوس موتانس استفاده شود .   یافته­ها: نتایج نشان دهنده­ اثر مهاری عصاره­ متانولی با mg/ml 25= MIC ، عصاره­ اتانولی با mg/ml 13/3= MIC و عصاره­ استونی با mg/ml 56/1= MIC بر روی رشد و تکثیر استرپتوکوکوس موتانس بود. عصاره­ آبی گیاه استویا و استویوساید باعث مهار رشد سویه­ مذکور نگردیدند.   مواد و روش­ها: در این تحقیق عصاره­ آبی و استونی گیاه استویا به روش خیساندن و عصاره­ متانولی و اتانولی به وسیله­ دستگاه سوکسله تهیه گردید. گلیکوزید استویوساید از کشور مالزی خریداری شد. برای بررسی عملکرد ضد باکتریایی عصاره ها و استویوساید از روش انتشار در چاهک با استفاده از اندازه گیری قطر هاله عدم رشد و تعیین حداقل غلظت مهاری توسط روش میکروبراث دایلوشن استفاده شد. سپس تأثیر استویوساید و هر کدام از عصاره ها با هم و نیز با آنتی بیوتیک پنی سیلین با روش آنالیز واریانس مقایسه گردیدند.

Title: تأثیر یک دوره تمرین ورزشی ترکیبی بر سطوح استراحتی لپتین پلاسما و برخی عوامل هورمونی در مردان دارای اضافه وزن

http://yafte.lums.ac.ir/article-1-1591-fa.html

مقدمه: هدف از این تحقیق مطالعه تاثیر یک دوره تمرین ورزشی ترکیبی بر سطوح استراحتی لپتین پلاسما و برخی عوامل هورمونی در افراد دارای اضافه وزن بود.   مواد و روش­ها: آزمودنی های این تحقیق 30 مرد دارای اضافه وزن با 29 BMI≥ دامنه سنی 22-42 سال بودند که به صورت تصادفی به دو گروه تجربی (15 نفر) و کنترل (15 نفر) تقسیم شدند. برنامه تمرین ترکیبی مقاومتی هوازی شامل 8 هفته تمرین و هر هفته 3 جلسه و هر جلسه شامل 12- 10 تمرین قدرتی ایستگاهی با شدت 75-70 درصد 1RM در چهار هفته اول و در چهار هفته دوم با شدت 80-75 درصد 1RM درنظرگرفته شد و در انتها 10 دقیقه تمرین دویدن هوازی با شدت 75-70 درصد MHR در چهار هفته اول و در چهار هفته دوم 13 دقیقه با شدت 80- 75 درصد MHR بود .   بحث و نتیجه­گیری : به طورکلی به نظر می رسد، کاهش لپتین در اثر یک دوره تمرین ترکیبی بیشتر با کاهش چربی بدنی، وزن و BMI در ارتباط است تا در اثرتغییرات هورمون تستوسترون یا کورتیزول. بنابراین، در مقایسه با بیشتر تحقیقات مربوط به تمرینات با وزنه که عدم تغییر در لپتین را گزارش کرده اند، شیوه تمرین ترکیبی راهبرد مؤثرتری به نظر می‌رسد.   یافته­ها: نتایج این تحقیق نشان داد میانگین تفاضل مقدار لپتین گروه تجربی در مقایسه با گروه کنترل کاهش معنی داری را نشان داده است (048/0 P= ). همچنین، 8 هفته تمرین ترکیبی باعث کاهش معنی دار BMI و وزن (05/0 P≤ ) آزمودنی‌ها شد. میانگین تفاضل مقدار تستوسترون و کورتیزول گروه تجربی در مقایسه با گروه کنترل تفاوت معنی داری را نشان نداد. میانگین تفاضل مقدار انسولین گروه تجربی در مقایسه با گروه کنترل کاهش معنی داری را نشان داد (02/0= P ). مقدار نسبت هورمون تستوسترون به کورتیزول ( T/C ) در گروه تجربی افزایش اندکی را نشان داد.

Title: بررسی میزان شیوع ژن بتالاکتاماز VEB1 درسویه های سودوموناس آئروژینوزای جدا شده از ایزوله های بیمارستانی با مقاومت چندگانه

http://yafte.lums.ac.ir/article-1-1606-fa.html

بحث و نتیجه­گیری : فراوانی سویه های تولیدکننده ژن بتالاکتاماز VEB1 در سویه های بیمارستانی روند روبه رشدی داشته است که این امرلزوم توجه بیشتر مراکز بهداشتی را در مورد تجویز دارو نشان می دهد. همچنین پیشنهاد می­گردد فراوانی سویه های مولد بتالاکتاماز به منظور جلوگیری از انتشار این ژن ها، توسط روش های مولکولی و سریع بررسی گردد.   مواد و روش­ها: سویه های سودوموناس آ ئروژینوزا از نمونه های بالینی جمع آوری و آزمایش­های استاندارد به منظور شناسایی سویه ها انجام شد. میزان مقاومت سویه ها به آنتی بیوتیک های مختلف بررسی و سپس از تکنیک واکنش زنجیره ای پلیمراز (PCR) برای ردیابی ژن VEB1 استفاده شد.   یافته­ها: بیشترین مقاومت به آنتی بیوتیک های آمیکاسین و سفوتاکسیم با 65% و 62% و کمترین مقاومت به آنتی بیوتیک­های پیپراسیلین (48%) و ایمی پنم (55%) مقاومت مشاهده شد. تعداد 38 سویه (60%) تولیدکننده ژن بتالاکتاماز VEB1 بودند.   مقدمه: بتالاکتامازها از آنزیم های تولید شده توسط پاتوژن های فرصت طلب و شایعی مانند سودوموناس آئروژینوزا هستند که کرباپنم ها را مهار و قادر به هیدرولیز طیف وسیعی از بتالاکتام ها هستند و به راحتی می توانند به سایر باکتری­ها نیز منتقل شوند. بنابراین هدف از این مطالعه، جداسازی باکتری سودوموناس آئروژینوزا و شناسایی سویه های واجد ژن کدکننده بتالاکتاماز VEB1 در نمونه های بالینی در شهر اصفهان با استفاده از تکنیک واکنش زنجیره ای پلیمراز (PCR) است .

Title: اولویت‌بندی شاخص‌های عملکردی حیطه کیفیت - اثربخشی بیمارستان‌های عمومی با استفاده از تکنیک تحلیل سلسله مراتبی (AHP)

http://yafte.lums.ac.ir/article-1-1607-fa.html

مقدمه: کیفیت مراقبت‌های بهداشتی عبارتست از درجاتی از خدمات ارائه شده به افراد و جوامع که احتمال نتایج مطلوب را افزایش داده و مطابق با دانش حرفه‌ای روز باشد. به منظور سنجش کیفیت خدمات ارائه شده در بیمارستان نیاز به شناسایی شاخص‌ها و استاندارد‌های عملکردی است، چرا که این شاخص‌ها ، اساس ساختار ارزیابی بیمارستانی را تشکیل می‌دهند. این مطالعه با هدف تعیین شاخص‌های عملکردی بیمارستان‌های عمومی در حیطه کیفیت - اثربخشی و اولویت بندی این شاخص‌ها انجام پذیرفته است. بحث و نتیجه‌گیری: شاخص‌های عملکردی مربوط به کیفیت - اثربخشی از جایگاهی مهم در ارزیابی‌های نظام سلامت و سازمان بیمارستان، برخوردار است. با استفاده از ارزشیابی کیفیت خدمات در مراکز بهداشتی‌درمانی می‌توان مشکلات و نواقص را بررسی کرد و با تجزیه و تحلیل اطلاعات در جهت کاهش آنها کوشید. یافته‌ها: در حیطه کیفیت - اثربخشی 73 شاخص عملکردی از بررسی متون مختلف حاصل شد و از این میان شاخص‌های عملکردی نرخ بروز عفونت بیمارستانی (100%)، میزان شیوع حوادث بیمارستانی (72%)، میزان خالص مرگ و میر بیمارستانی(63%) و درصد رضایت بیماران (53%) به عنوان شاخص‌های عملکردی اولویت‌دار برگزیده شد. مواد و روش‌ها: این مطالعه، یک مطالعه توصیفی- کیفی و از نوع کاربردی بوده که از طریق بررسی سیستماتیک متون و دریافت اطلاعات از صاحب‌نظران بیمارستان از طریق پرسشنامه صورت پذیرفته است. به منظور اولویت‌بندی شاخص‌های عملکردی حیطه کیفیت - اثربخشی از تکنیک تحلیل سلسله مراتبی (AHP) استفاده شده و نتایج بدست آمده با نرم افزار Expert Choice 11 تحلیل مدیریتی شده است.
